# A Medico-Legal Review of the Case of Patrick Maude; Who Was Convicted for Murder and Executed at Newark, N. J.

**Published:** 1860-03

**Authors:** Charles F. J. Lehlbach

**Affiliations:** New York


*A Medico-Legal Review of the Case of Patrick Maude; who was convicted for murder and executed at Newark, N. J.—The Plea of Insanity.* By Charles F. J. Lehlbach, M. D., of New York.

On the 28th day of May, 1859, Patrick Maude shot his sister, Mary Ann Turbett, in the City of Newark, N. J. He was tried for this crime in the October term of the Court of Oyer and Terminer, of the County of Essex, convicted, sentenced to death, and executed on January 12th, 1860. The district Attorney, Mr. Cortlandt Parker, was assisted by the Hon. Wm. L. Dayton.

The plea of insanity entered into the defence, and as the case has excited a great deal of discussion in the public prints, and is now, as it probably will be for some time to come, the subject of considerable controversy, we believe that a short review of its leading facts, with the conclusions to be drawn therefrom, will not be inappropriate to your readers.

A few days before the murder was committed, Patrick Maude had escaped from the State Lunatic Asylum. He had been placed there by order of the same court before which his last trial took place; a Commission of Physicians, appointed by that court, having declared him insane, when on trial for an assault upon his wife, and an attempt to take her life.

The circumstance of Maude's escape from the Asylum, his journey from Trenton to Newark, New York, (where he procured a pistol,) and his return to Newark by way of Elizabethport, of his arrest after the committal of the crime, it is not necessary to detail here. Suffice it to say, that like most insane, who have escaped their confinement or are bent on criminal purposes, he acted cautiously and with a considerable amount of cunning and logical reasoning, to escape capture and detection.

Of the early history of Maude, whether there was any hereditary tendency to insanity in his family, what were his habits, his propensities, inclinations and previous diseases, little is known that would be interesting in this connection. Our information is limited chiefly to the following facts:

That he was born in Dublin County, Ireland, received a pretty good education, worked in chemical factories, distinguished himself in his art, so as to occupy positions as overseer, etc., emigrated to the United States, found employ in the chemical works in Newark, and other places; was married; got in the habit of drinking at times to excess; began to act strangely and wildly; became possessed of the idea of a conspiracy against him; finally made the attack upon his wife; was placed upon trial; acquitted, because judge, jury and prosecution saw that he was insane; was sent to the State Asylum whence he escaped; shot his sister; was captured, tried, convicted and executed.

His disposition is described by those who knew him for the last five or six years, as quarrelsome and wild, but not always so. He had intervals in which his conduct was all that could be desired.

### *Evidence of Maude's Insanity*

1. The crime for which Maude was executed, was committed as has been already stated, a few days after his escape from the lunatic asylum. Our personal acquaintance with the gentlemen who composed the medical commission examining into his mental sanity, on the occasion of his first trial, under the direction of the court; their high scientifie attainments, and large experience, precludes the idea than they would have destined him to a residence in the insane asylum, had they not been fully convinced of the unsoundness of his mind. Nor do we believe that the able Superintendent of the Asylum would have retained him a day, had he been convinced that Maude was sane and that thus he could be either let free, or placed in the hands of other public officers. These facts afford strong presumptive evidence of Maude's insanity.

2. There is no doubt, that Maude was subject to strong hallucinations, both sensual and intellectual, if we are permitted to make that distinction; using the term 'sensual hallucinations,' for impressions received by the brain apparently through the senses, where there is not the slightest *reality* of the supposed objects of the impression; and applying the term "intellectual hallucinations" to that condition, where the brain originates perceptions, ideas, thoughts and conclusions, that are based, either upon the sensual hallucinations, the belief in their reality, or appear to be entirely spontaneous. How far the condition of Maude's brain that give rise to these hallucinations, was caused by real occurences, by violent emotional shocks, the repeated excitement of which led to permanent disease of the brain, or whether the disease commenced *first* in the brain and manifested itself in the morbid exaggeration, the delusive and hallucinatory view which he took of the relations between himself, his family and his fellow-men, are questions, the exact solution of which, although it would reveal the *etiology* of the disease, still cannot be regarded as a vital link in the chain of evidence; being a question of psychical pathology, rather than medical juriprudence. The *main* question in a *medico-legal point of view*, is, *whether these hallucinations did exist*, and to an examination of this point we invite the reader, by adducing the following extracts from the medical evidence given in the case.

Dr. G. Grant had examined Maude, May 30th, 1859, in the country jail; his eyes were fixed, unwavering; pulse 100, full and hard; pupils dilated; his memory appeared to be good; he stated that he was born in Dublin County, Ireland, and was 44 years of age; on examining as to his motives (this was two days after the commission of the crime,) he talked somewhat wildly, accursed Father C—— as the source of all his trouble; said that his sister and wife laid in with Father C—— to ruin him; "my life is embittered, my peace destroyed, and thus by their combined annoyance to me, I was rendered desperate, and though not insane, I was not entirely sane *because of this danger and annoyance to which I was subjected by them.* I am so distressed by their acts, that *I constantly hear them*; something appears to my brain (placing his hand upon the back of his head) always, except when I sleep *I hear them;* I am not insane; I do not wish to make any such plea (speaking very excitedly)." Upon being told what without such plea, the punishment would be, he said "death is better than life; I am so distracted and annoyed that I do not care about living;" said he heard noises, and talking of his enemies, and had constant ringing in his ears.

Dr. A. N. Dougherty, physician to the County Jail; I knew the defendant; had seen him frequently, *prior to his being sent to the asylum;* I have seen him several times during his present incarceration; I was requested by the court to examine with Drs. Smith and Pennington into his state of mind; *found that he was laboring under the delusion that Callan, his wife, and Mrs. Turbett had made a conspiracy to take his property; he complained that he heard their voices overhead in a cell, conspiring against him, that he was the subject of witchcraft, practiced upon him by these parties* ........... Dr. D. considers his present condition of mind the same as when he was sent to the asylum.

Dr. J. Henry Clark, gives his opinion that he considers Maude insane; looks upon him as laboring under a hallucination which has directed his conduct in the past.

Dr. Lyndon A. Smith made an examination of Maude, and thought *that he labored under a delusion;* he spoke of *conspiracy against him to get his property and destroy it;* Dr. S. considers him as *still laboring under that delusion;* thinks him a remarkably strong-minded man in every other respect; should judge that Maude knew right from wrong, as well as any one.

Dr. H. H. Tichenor: I visited Maude the second day after his arrest; think it was on Monday; I examined his pulse; saw no symptoms of disease of the brain. He appeared a litttle excited, and his pulse was somewhat accelerated. I saw him on the night of his arrest, and he exhibited no signs of insanity. He said that if the person who shot Mary Turbett had shot Father Callan and a few others, the city would not have been disgraced by it. He complained of being sent to the Lunatic Asylum instead of to the State Prison for a limited time. He spoke about his having been employed at the Chemical Works. Said his wife had allowed his property to be sold, and that the powder had been in his pocket since he shot the old woman. Saw no delusion about him at that time, and did not think him insane. When I visited him at the jail my opinion was somewhat changed. His thoughts were collected and his memory good. He said, after some general conversation, *that there was a heavy conspiracy*

*against him, and that he could hear the voices of the conspirators in the cell above him. He said he had a roaring in his ears; said he knew for what he was imprisoned· He said the conspirators intend to bring him to the gallows, and he supposed they would do it.* His property was gone and his wife was not of much value to him. The conversation was conducted mostly by questions and answers. I put questions to determine his delusion, if he had any. I thought he was capable of judging between right and wrong. He made some remark about the injustice of the laws, and the manner in which he had been treated. In my last interwiew I formed an opinion that he was laboring under a delusion which might to some extent lesson his responsibility.

Dr. Buttolph, superintendent of the State Lunatic Ayslum, at Trenton, testified that Maude was apparently sane on all subjects, *except this conspiracy;* that it was necessary oftentimes to keep him in close confinement *on account of his excitement on this subject;* Maude's conversation while at the asylum, on all subjects, except this alleged conspiracy was rational, Dr. B. thinks that he reasoned with himself that *if he kill his conspirators he would be acting in self-defence, and would be justifiable;* he frequently threatened the lives of his *conspirators*—sometimes as satisfaction, sometimes as revenge; *his case was a species of monomania;* Dr. B. would consider his insanity as consisting of his delusion on this conspiracy, and the overaction of his revengeful feelings for being sent to the asylum; the state of Maude's mind, while in the asylum, led Dr. B. to consider him a partially insane man; his opinion was that he would never be otherwise; his partial insanity was owing to the delusion on one subject, the conspiracy against him; he always appeared rational on all other subjects.

3. But aside from the opinions above expressed by the medical witness, as experts, their simple statements as intelligent men, are strong proofs of Maude's insanity. Dr. Dougherty states that Maude believed himself under the influence of witchcraft, practiced upon him by his conspirators. (Intellectual hallucination;) This is not a mere superstitious belief—but became in him a hallucinatory reality, when he hears his enemies converse and plot above his cell. (Sensual hallucination.)

These facts, with the incontrovertible evidence that Maude believed, lived, and, as the progress of the case shows, *died*, in the hallucinatory idea of a conspiracy against him, are of the utmost importance. A man who hears his enemies loudly and often angrily converse, when all is quiet; who believes in the reality of these hallucinations; who has noises in his ears, and a tearing pain in his back; who believes himself the victim of witchcraft and inquisitorial conspiracy, must be considered as insane, and his acts cannot be looked upon as the result of free agency.

(4.) Maude possessed that characteristic of insanity which is always present when the disease is hallucinatory, namely, *a strong, even passionate aversion against being coxsidered insane*, so prominent was this trait in his case, so shrewd, *apparently*, and logical was his reasoning, that one of the chief grounds upon which the prosecution rested its demand for a verdict of guilty was, "that it would be strange to acquit a man on the plea of insanity, who evinced the acumen and skill that he did on the defence."—(Mr. Dayton's speech.)

[To be Continued.]

